# The Association Between Elevated Progesterone Level on Day of hCG Trigger and Live Birth Rates in ART Cycles: A Single Centre Observational Study

**DOI:** 10.18502/jri.v21i4.4333

**Published:** 2020

**Authors:** Shahin Robati, Wiam Saab, Montserrat Durán-Retamal, Wael Saab, Efstathios Theodorou, Suzanne Cawood, Paul Serhal, Srividya Seshadri

**Affiliations:** 1- Institute for Women’s Health, Faculty of Population Health Sciences, University College London, London, United Kingdom; 2- Department of Obstetrics and Gynaecology, The American University of Beirut Medical Centre, Beirut, Lebanon

**Keywords:** ART, hCG trigger, *In vitro* fertilization (IVF), Live birth rate (LBR), Ovarian stimulation, Progesterone elevation

## Abstract

**Background:**

The advent of ovarian stimulation within an *in vitro* fertilization (IVF) cycle has resulted in modifying the physiology of stimulated cycles and has helped optimize pregnancy outcomes. In this regard, the importance of progesterone (P4) elevation at time of human chorionic gonadotrophin (hCG) administration within an IVF cycle has been studied over several decades. Our study aimed to evaluate the association of P4 levels at time of hCG trigger with live birth rate (LBR), clinical pregnancy rate (CPR) and miscarriage rate (MR) in fresh IVF or IVF-ICSI cycles.

**Methods:**

This was a retrospective cohort study (n=170) involving patients attending the Centre for Reproductive and Genetic Health (CRGH) in London. The study cohort consisted of women undergoing controlled ovarian stimulation using GnRH antagonist or GnRH agonist protocols. Univariate and multiple logistic regression analyses were used to evaluate the association of clinical outcomes. Differences were considered statistically significant if p≤0.05.

**Results:**

As serum progesterone increased, a decrease in LBR was observed. Following multivariate logistical analyses, LBR significantly decreased with P4 thresholds of 4.0 *ng/ml* (OR 0.42, 95% CI:0.17–1.0) and 4.5 *ng/ml* (OR 0.35, 95% CI:0.12–0.96).

**Conclusion:**

P4 levels are important in specific groups and the findings were statistically significant with a P4 threshold value between 4.0–4.5 *ng/ml*. Therefore, it seems logical to selectively measure serum P4 levels for patients who have ovarian dysfunction or an ovulatory cycles and accordingly prepare the individualized management packages for such patients.

## Introduction

The Human Fertilisation and Embryology Authority (HFEA) data shows the live birth rate (LBR) for *in vitro* fertilization (IVF) is only 26.6% for all ages (1). Over the years, considerable research has been conducted on the endocrine physiology in IVF cycles in order to ensure high LBRs and achieve good pregnancy outcomes.

The normal menstrual cycle comprises follicular and luteal phase. The cycle commences from day 1 of menstruation until ovulation, around day 14. During this time, the hypothalamus releases GnRH, which stimulates the synthesis and release of Follicle Stimulating Hormone (FSH) and Luteinizing Hormone (LH) from the anterior pituitary gland. In turn, FSH stimulates the growth and maturation of ovarian follicles within the ovary. The luteal phase commences after ovulation. During this phase, the ruptured follicle forms the corpus luteum, which produces high levels of P4 and is responsible for the maintenance and decidualization of the endometrium. Recently, this has been assumed to be related to prolonged pituitary activity, moderated by P4 and LH (2). Following a surge in LH and high levels of P4, the ovarian follicle releases the mature oocyte. The process leading to development of a mature oocyte within a normal menstrual cycle is therefore important.

The importance of P4 elevation on the day of hCG trigger in assisted reproductive technology (ART) cycles was first published over 20 years ago in 1991 (3). Over the past decade, the presence of P4 elevation at the time of hCG administration within an IVF cycle has become increasingly studied and there have been conflicting reports over the association of progesterone elevation (PE) with the live birth rate (LBR) and clinical pregnancy rate (CPR) (4–6). Up until 2006, a systematic review of published reports showed that PE was associated with a low CPR (7). A subsequent systematic review and meta-analysis in 2012 showed that PE on the day of hCG trigger was significantly associated with a lower CPR (8).

The most recent systematic review and meta-analysis showed an estimated 10% decrease in the probability of a pregnancy in the presence of P4 elevation (4); however, no adverse effect was noted in frozen-thawed and donor/recipient cycles. Although no further systematic review or meta-analyses have been published since, other studies have tried to validate these findings with LBR as an end-point measure (5, 9). As a result, studies have focused on both prevention and management of P4 elevation at the time of hCG trigger (10, 11). Unfortunately, limited high quality studies currently exist.

It is not well known if additional variables occur which would help identify patients with P4 elevation on the day of hCG administration (12). Determining these baseline characteristics, together with an attempt to quantify the relationship between P4 elevation at the time of hCG administration and pregnancy outcomes amongst different ovarian protocols, forms an integral part of our aims and objectives. The primary aim of this study was to evaluate the association of P4 elevation at the time of hCG administration with LBR, CPR and MR in fresh IVF/ICSI (Intracytoplasmic sperm injection) cycles within our study population. Our secondary aim was to carry out subgroup analyses of the data by analyzing associations between P4 elevation at the time of hCG trigger and baseline patient characteristics, as well as analyzing potential confounding variables.

To the best of our knowledge, this is the first study performed in the UK regarding P4 elevation at the time of hCG trigger. The findings are important for local practice and potential wider implications.

## Methods

### Study design:

This non-interventional, retrospective, observational single-center cohort study was conducted in women undergoing treatment for infertility at a single tertiary IVF center at the Centre for Reproductive and Genetic Health (CRGH), UK. This study was an analysis of fresh IVF or IVF-ICSI cycles in women undergoing ovarian stimulation using GnRH-antagonist or GnRH-agonist protocols between January 2015 and September 2016. The follow-up time of patients was up to week 10 of gestation and live birth outcomes were reported as per the HFEA UK legislation.

### Study population:

A total of 170 women undergoing assisted reproduction were included in the study. Women underwent fresh IVF cycles, which included ICSI, in which hCG was administered for triggering final oocyte maturation. The data was collected from medical records and computerized databases within a 21 month period. No inclusion or exclusion criteria were applied on baseline demographics, with the intent to show the wide variety of patients seen within the clinical setting.

### Inclusion criteria:

All patients between ages 20–45 years undergoing a fresh IVF or fresh IVF-ICSI cycle with a GnRH-antagonist or GnRH-agonist protocol at CRGH during the study time period were included. The cases with advanced endometriosis and adenomyosis were included as they were given the ovarian stimulation protocol that optimizes their outcomes. Also, cases with underlying medical conditions such as thrombophilia were included and managed by the local haematologist. Progesterone level was measured on all study subjects on day of hCG administration.

### Exclusion criteria:

Patients who had P4 levels measured on days other than the day of hCG administration were excluded from the study. Patients who underwent pre-implantation genetic screening (PGS) or pre-implantation genetic diagnosis (PGD), as well as oocyte freezing, embryo cryopreservation or a natural IVF cycle were also excluded. Patients with uterine anomalies were automatically excluded, as 3D scanning and saline infusion sonohysterography (SIS) was a screening tool to rule out abnormalities prior to commencing their fresh cycle. In total, 77 patients were excluded from the study (31.1% exclusion rate), leading to a study sample of 170.

### Protocols for controlled ovarian stimulation:

Selecting the most appropriate ovarian stimulation protocol and type of gonadotrophin, principally recombinant FSH, was individualized according to patient characteristics and clinical judgement. Buserelin (Suprefact^®^; Hoechst, Germany) was used for pituitary down-regulation. This was performed with either a GnRH antagonist short protocol, known as the Cetrotide^®^ protocol (n=52, 30.6%), or a GnRH agonist long protocol, which uses either the mid-luteal protocol (MLP) (n=60, 35.3%), or the sub-optimal mid-luteal protocol (SO-MLP) (n=58, 34.1%). In our study population, 30.6% received the short protocol and 69.4% received the long protocol. Follicular growth was deemed satisfactory once at least three follicles had reached a diameter of 16 *mm*. Cetrotide^®^ and FSH were then stopped, with subsequent administration of 10,000 international units (IU) of hCG (Pregnyl^®^; Organon, Australia or Ovitrelle^®^; Geneva, Switzerland) given by intramuscular (IM) injection to trigger final oocyte maturation. After 34–36 *hr*, oocyte retrieval and follicular aspiration was achieved by Transvaginal Ultrasound Scan (TV-USS).

### Embryo transfer:

Approximately 34–36 *hr* after hCG trigger, oocyte retrieval and follicular aspiration was achieved by transvaginal ultrasound scan. The retrieved oocytes were incubated for several hours in fertilization medium. Embryo grading was classified according to the Association of Clinical Embryologists (ACE) and the UK National External Quality Assessment Service (UK-NEQAS) (13). Embryo transfers were either performed at the cleavage stage (Day 2 to 3) or the blastocyst stage (Day 5 to 6). Up to 2 embryos were replaced based on patient’s age, previous history, together with the embryo developmental stage and embryo quality. Additional embryos, which were of good quality, were cryopreserved for future frozen-thawed cycles.

### Luteal-phase support:

Following oocyte collection, patients received progesterone for luteal phase support. This was administered via the vaginal route as intravaginal gels (Crinone^®^; Darmstadt, Germany). Patients were also commenced on estradiol valerate (Progynova^®^; Berlin, Germany) until a clinical pregnancy was confirmed. P4 levels did not affect the management of these cycles, with no cancellations due to progesterone elevation (PE).

### Hormone measurements:

Serum P4 concentrations were measured on the morning of hCG administration as well as during ovarian stimulation. Intra-assay and inter-assay precision, expressed as coefficients of variation for serum P4, was <3% and 5%, respectively. Electro-chemiluminescence immunoassay (ECLIA) was used to measure serum P4 concentrations, with the platform Roche E170. The lower sensitivity limit was 0.09 *nmol/l* and the functional sensitivity was 0.48 *nmol/l*. Samples measuring FSH, E2 and β-hCG were test-ed using an automated immunoassay analyzer (El-ecsys^®^; Roche Diagnostic, Mannheim, Germany).

### Statistics:

For continuous variables, the mean, standard deviation (SD) and the standard error of the mean (SEM) were calculated. Comparing continuous variables between groups was performed using the Student’s t-test or the Mann-Whitney U-test, which was dependant on whether the data was normally distributed. For categorical variables, the Chi-squared test was used as the appropriate measure. To identify the P4 threshold for a detrimental effect on LBR, CPR and MR, the odds ratio (OR) and 95% confidence interval (CI) for these clinical outcomes were calculated. Univariate and multivariate logistic regression was also used for further measurements. Statistical analysis on all 170 patients was performed using the Statistical Package for Social Sciences (SPSS^®^) version 24 for Mac (SPSS^®^; Chicago, USA). A p-value of ≤0.05 was considered statistically significant for all statistical tests.

## Results

### Baseline characteristics:

In brief, 170 patients underwent fresh IVF/ICSI cycles. The average age of participants was 35.7 years (Range, 20–45 years). The serum P4 concentration level (Mean± SEM) was 2.57±0.15 (Range, 0.5–11.8 *ng/ml*). The median was 2.1, which was used as the cut-off value. The median was chosen compared to the mean, as the data for P4 was skewed and asymmetrical. The patients’ data and cycle characteristics of the sample analyzed in our study population are presented in table 1.

**Table 1.** Baseline characteristics and ovarian stimulation characteristics

**Parameter ^a^**	**Serum progesterone on day of hCG trigger**		**p-value ^b^**	**Total**

	**<2.1 (n=83)**	**≥2.1 (n=87)**		
**Age (years)**	36.41 (4.7)	34.98 (3.6)	0.027	35.70 (4.2)
**BMI (*kg/m^2^*)**	25.13 (5.2)	23.37 (3.8)	0.153	24.25 (4.5)
**AMH (*pmol/l*)**	19.3 (12.6)	16.4 (11.4)	0.199	17.85 (12.0)
**Number of oocytes collected**	11.28 (4.6)	11.16 (5.0)	0.866	11.22 (4.8)
**Endometrial thickness**	10.9 (2.5)	10.34 (2.2)	0.139	10.62 (2.3)

a) All values are presented as mean (SD),

b) Student's t-test or Mann-Whitney U test for differences between normal and elevated progesterone groups (P4 cut-off 2.1 *ng/ml*)

### Association of P4 elevation with LBR:

**Figure 1.**
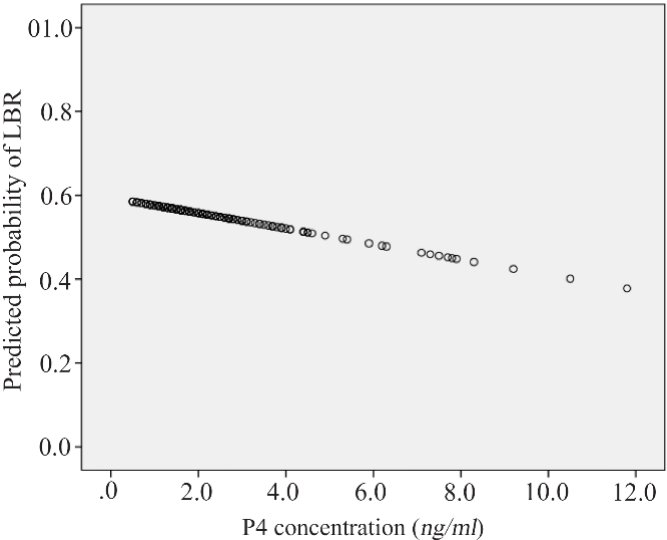
Predicted probability in LBR per serum P4 value

The overall LBR in our study population was 54.7% (OR: 0.93, 95% CI: 0.79–1.09; p=0.357). While there was a 7% decrease in the live birth rate per 1.0 *ng/ml* P4 increase, this was not statistically significant. As the serum P4 value increases, a trend for a lower LBR could be observed, without reaching statistical significance. The predicted probability of a live birth per P4 (*ng/ml*) level is presented in figure 1; however, in order to validate the robustness of this trend, the analysis was repeated for different P4 thresholds. They were categorized with increasing increments of 0.5 *ng/ml* up to 5.0 *ng/ml*. Subsequent multivariate logistical regression analyses were performed to assess the correlation of P4 with LBR, depicted in table 2. The results showed that live birth rates significantly decreased with P4 thresholds of 4.0 *ng/ml* (OR 0.42, 95% CI: 0.17–1.0; p=0.05) and 4.5 *ng/ml* (OR 0.35, 95% CI: 0.12–0.96; p=0.041), whereas the relationship between the LBR and P4 levels was not statistically significant at other thresholds. With a cut-off value of 4.5 *ng/dl*, there is a 65% decrease in the LBR if P4 is ≥4.5 *ng/ml*, although the wide 95% confidence interval should be noted. In addition, subgroup analysis showed no significant difference (OR 1.32, 95% CI: 0.72–2.43) with a P4 cut-off of 2.1 *ng/ml* (Median value).

**Table 2.** Association of P4 elevation with live birth rates for different thresholds

**P4 Threshold (*ng/ml*)**	**p ^a^**	**OR**	**95% CI**	

			**Lower**	**Upper**
**1**	0.526	1.37	0.53	3.57
**1.5**	0.905	0.96	0.49	1.87
**2**	0.643	1.15	0.63	2.11
**2.5**	0.912	0.97	0.52	1.79
**3**	0.227	0.66	0.33	1.3
**3.5**	0.182	0.60	0.28	1.27
**4**	**0.050**	0.42	0.17	1.00
**4.5**	**0.041**	0.35	0.12	0.96
**5**	0.248	0.53	0.18	1.56

a) Analysis performed using Mann-Whitney U test. Values in bold show statistical significance

### Variables associated with LBR:

Several variables were recorded for the study population (n=170). These include age (n=170, 100%), BMI (n=58, 34.1%), AFC (Antral follicle count) (n=145, 85.3%), AMH (Anti-Müllerian hormone) (n=115, 67.6%), number of oocytes collected (n=164, 96.4%) and endometrial thickness (n=156, 91.8%). The only variable significantly associated with LBR was age (OR 0.82, 95% CI 0.69–0.97; p= 0.023) in table 3.

**Table 3.** Effect of variables associated with LBR when PE ≥2.1 *ng/ml*

**Confounding variables**	**p ^a^**	**OR**	**95% CI**	

			**Lower**	**Upper**
**P4**	0.397	0.83	0.54	1.28
**AMH**	0.704	0.98	0.89	1.08
**Age**	**0.023**	0.82	0.69	0.97
**BMI**	0.904	1.01	0.86	1.19
**AFC**	0.998	1	0.87	1.16
**Number of oocytes collected**	0.315	1.09	0.93	1.27
**Endometrial thickness**	0.32	0.86	0.64	1.16

a) Student's t-test or Mann-Whitney U test. Values in bold show statistical significance

### P4 sensitivity and specificity related to LBR:

**Figure 2.**
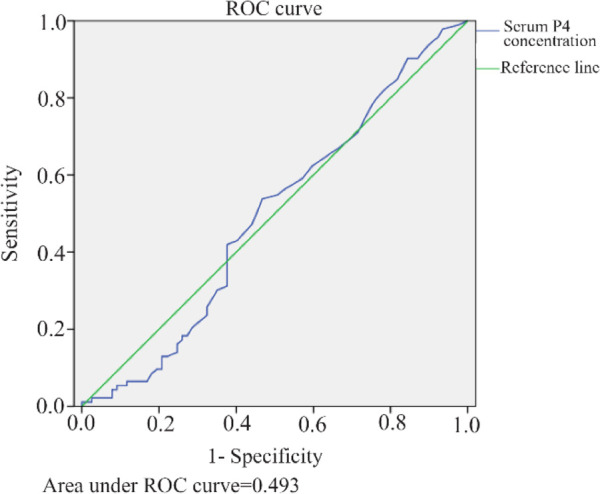
Area under the ROC curve for serum P4 concentration *in* LBR

To further assess the predictive value of serum P4 concentration on the day of hCG trigger of a successful live birth, an ROC (Receiver-operating characteristic) curve analysis was performed (Figure 2). The AUC (Area under the curve) was 0.493, indicating the correlation of P4 concentration and LBR is less than 50% accurate and no better than chance. The median P4 value was 2.1 *ng/ml*. This cut-off value had a sensitivity of 50.6% and a specificity of 54.5%.

### Association of P4 elevation and CPR:

There were 110 clinical pregnancies from 170 fresh embryo transfer cycles, with CPR of 64.7% among all three protocols. P4 elevation did not show any significant correlation with a CPR (OR 0.89, 95% CI 0.75, 1.04; p=0.139); however, the trend appears to be an 11% reduction in odds of a clinical pregnancy per 1 *ng/ml* increase of P4.

### Variables associated with CPR:

Following further subgroup analysis, age in the Cetrotide^®^ protocol was the only significant variable in predicting a clinical pregnancy (OR 0.81, 95% CI: 0.67–0.99). Similar results were seen in the LBR cohort, whereby age was the only significant variable.

### Association of P4 elevation with MR:

There were 29 miscarriages from 170 patients in our study population. The overall MR was 17.1% (OR: 0.92, 95% CI: 0.73–1.16). Although there was an 8% decrease in odds of a miscarriage per unit increase of P4, this was not statistically significant and conversely may show a 16% increased risk of a miscarriage.

### Association between different protocols and LBR:

The MLP protocol showed the highest LBR (58.3%), followed by the SO-MLP and Cetrotide^®^ protocol (53.4% and 50.9%, respectively). Live birth rates were not significantly different between protocols (p=0.571). Further subgroup analyses showed no significant difference in LBR with and without PE (≥2.1 *ng/ml*) when multivariate analysis was performed.

## Discussion

To the best of our knowledge, this is the first UK study evaluating the association of P4 elevation on the day of hCG administration and LBR, CPR and MR. This brings novelty and originality to the study.

On the basis of the findings, increased serum P4 concentrations on the day of hCG trigger is associated with a reduced probability of a live birth after fresh embryo transfer. This finding was present in all P4 threshold groups, except a P4 threshold value of 1.0 *ng/ml* and 2.0 *ng/ml*. Nonetheless, the finding was statistically significant with P4 thresholds of 4.0 *ng/ml* and 4.5 *ng/ml*. This is consistent with the most recent systematic review and meta-analysis, which involved 63 studies in over 55,000 fresh IVF/ICSI cycles (4); however, the achievement of pregnancy was defined as an ongoing pregnancy, live birth or clinical pregnancy, whereas our study separated these pregnancy outcomes. More studies should be performed to further evaluate this finding. Although lower P4 thresholds were observed in the meta-analysis, this may be explained by using different global P4 assays compared to the ones in UK. There should be a standardized progesterone level at which a value should be measured for freeze-all cycles.

The MLP ovarian stimulation protocol showed the highest LBR (58.3%); however, this was not significant compared to the Cetrotide^®^ and SO-MLP protocol following multivariate analysis. Nonetheless, this is also consistent with a recent systematic review and meta-analysis, which compared women undergoing either a GnRH agonist or antagonist protocol within an IVF/ICSI cycle, with LBR being the primary outcome (14). The study showed no statistically significant difference between the two groups, although PE was not specifically accounted for. A more recent Cochrane review, which included 12 studies, 2 RCTs and studies over 2300 participants also confirmed no statistically significant association between protocols (15), but similarly did not specifically take PE into consideration.

The only significant variable in our study associated with a live birth was female age. The association between the reduction of both the number and quality of oocytes with increasing female age has long been established (16, 17), which is translated into a reduced LBR (18–20). Similarly, age was the only significant variable when compared to CPR.

Venetis et al. in 2007 concluded that "the best available evidence does not support an association between progesterone elevation on the day of hCG administration and the probability of clinical pregnancy in women undergoing ovarian stimulation (7)". Kolibianakis et al. only analyzed GnRH antagonist cycles (8). Venetis et al. used "probability of pregnancy achievement" as the primary endpoint, not live birth (4).

In terms of CPR, our study population showed that P4 elevation was also associated with a decreased trend in a clinical pregnancy, but failed to show statistical significance. On the other hand, a P4 cut-off value of 4.0 *ng/ml* was statistically significant for the long cycle protocol. The MR among pregnancies following ART has remained constant over the past two decades at around 15% (21). This is similar to our study population, which showed a MR of 17.1%.

The detrimental effect of P4 elevation on the predicted probability of a LBR in women undergoing fresh IVF/ICSI cycles was evident (P4 threshold ≥2.5 *ng/ml*) (Table 3). The maximal effect on the LBR was seen with a threshold between 4.0–4.5 *ng/ml* and diminished thereafter.

Our primary outcome measure was LBR, which is the principal outcome and the desire of couples attending the fertility clinic. This is often overlooked in many studies, which only report clinical pregnancy rates. In addition, the LBRs and CPRs in our study were high (54.7% and 64.7%, respectively). This is comparable to the recent systematic review and meta-analysis, which yields more statistical meaningful results (4). In order to be included in the current study, a patient had to undergo a fresh IVF or IVF-ICSI cycle during the study time period. Other inclusion and exclusion criteria were relatively non-stringent and minor. The study population in this cohort was heterogeneous. For example, one limitation of this study was that both GnRH-antagonist and GnRH-agonist protocols were analyzed together and this factor was not included in the multivariable logistic regression analysis. The retrospective and observational nature of the study means that the presence of bias in interpreting the data cannot be excluded. In addition, imbalances of the two groups within different P4 thresholds may confound the association of PE and LBR.

It seems that for women who undergo fresh ET, P4 elevation on the day of hCG trigger is associated with a decreased probability of a live birth and a clinical pregnancy. The results of the study indicate that both researchers and clinicians should utilize a multivariable approach, to reveal a more accurate estimate in the underlying association between PE and LBR, devoid of any significant confounding factors such as female age or number of oocytes retrieved. As a result, predicting the prognosis for fresh IVF/ICSI cycles will become more accurate to clinicians. More importantly, the decision on whether ET should be performed directly, or postponed to a frozen-thawed cycle should be encouraged. This is particularly relevant where elevated P4 has not compromised endometrial receptivity during a stimulated cycle. Nonetheless, in the absence of significant evidence confirming an effective way in managing women with PE from the current literature review, sound clinical judgment remains imperative in the decision-making process.

The results of this study would also help in better management and consultation with patients. In addition, serum P4 concentrations and factors related to the patient’s IVF cycle, such as the number of oocytes retrieved, a history of OHSS, or implantation failure secondary to previous P4 elevation, are important considerations. A detailed past medical history of patients is therefore important for individualizing an appropriate management plan. Our results merit further intensive studies and for raising the awareness of society, other fertility centres in the UK can be encouraged to address this clinically relevant and important research topic.

## Conclusion

Our research appears to be the first of its kind conducted in the UK, which suggests that elevated P4 levels at the time of hCG administration within a fresh IVF/ICSI cycle is associated with a decreased probability of a live birth and clinical pregnancy. The finding was statistically significant with a P4 threshold value between 4.0–4.5 *ng/ml*. A shift towards a freeze-thaw IVF cycle for women who have a P4 value above this threshold appears feasible. It is logical to selectively measure serum P4 levels for patients who have ovarian dysfunction or anovulatory cycles. Individualizing patient management and applying clinical judgement would help achieve the best clinical outcomes for infertile couples, which has an impact on many people’s lives.
